# SARS-CoV-2 Nsp14 binds Tollip and activates pro-inflammatory pathways while downregulating interferon-α and interferon-γ receptors

**DOI:** 10.1128/mbio.01071-25

**Published:** 2025-06-25

**Authors:** Naveen Thakur, Poushali Chakraborty, JoAnn M. Tufariello, Christopher F. Basler

**Affiliations:** 1 Department of Microbiology, Icahn School of Medicine at Mount Sinai 200769, New York, New York, USA; University of Florida College of Public Health and Health Professions, Gainesville, Florida, USA

**Keywords:** coronavirus, exonuclease, methyltransferase, Tollip, interferons, virus-host interactions

## Abstract

Severe acute respiratory syndrome coronavirus 2 (SARS-CoV-2) non-structural protein 14 (Nsp14) possesses an N-terminal exonuclease (ExoN) domain that provides a proofreading function for the viral RNA-dependent RNA polymerase and a C-terminal N7-methyltransferase (N7-MTase) domain that methylates viral mRNA caps. Nsp14 also modulates host functions. This includes the activation of NF-κB and downregulation of interferon alpha/beta receptor 1 (IFNAR1). Here, we demonstrate that Nsp14 exerts broader effects, activating not only NF-κB responses but also extracellular-signal-regulated kinase (ERK), p38, and Jun amino-terminal kinase (JNK) mitogen-activated protein kinase (MAPK) signaling, promoting cytokine production. Furthermore, Nsp14 downregulates not only IFNAR1 but also IFN-γ receptor 1 (IFNGR1), impairing cellular responses to both IFNα and IFNγ. IFNAR1 and IFNGR1 downregulation is via a lysosomal pathway and occurs in SARS-CoV-2-infected cells. Analysis of a panel of Nsp14 mutants reveals a consistent pattern. Mutants that disable ExoN function remain largely active, whereas N7-MTase mutations impair both pro-inflammatory pathway activation and IFN receptor downregulation. Innate immune modulating functions also require the presence of both the ExoN and N7-MTase domains, likely reflecting that the ExoN domain must be present to enable N7-MTase activity. We further identify multi-functional host protein Tollip as an Nsp14 interactor. Interaction requires the phosphoinositide-binding C2 domain of Tollip and sequences C-terminal to the C2 domain. Full-length Tollip or regions encompassing the Nsp14 interaction domain are sufficient to counteract both Nsp14-mediated and Nsp14-independent activation of NF-κB. Knockdown of Tollip partially reverses IFNAR1 and IFNGR1 downregulation in SARS-CoV-2-infected cells, suggesting the relevance of Nsp14-Tollip interaction for Nsp14 innate immune evasion functions.

**IMPORTANCE:**

Severe acute respiratory syndrome coronavirus 2 (SARS-CoV-2) non-structural protein 14 (Nsp14) both activates NF-κB, which promotes virus replication and inflammation, and downregulates interferon alpha/beta receptor 1 (IFNAR1), which can render infected cells resistant to the antiviral effects of IFN-α/β. Our study demonstrates that Nsp14 also activates MAPK signaling and downregulates IFN-γ receptor 1 (IFNGR1), causing broader impacts than previously recognized. Data from a panel of Nsp14 mutants suggest that a common underlying effect of Nsp14 may be responsible for its multiple innate immune activities. We further describe a novel interaction between Nsp14 and Tollip, a selective autophagy receptor. We show that Tollip expression downregulates Nsp14 activation of NF-κB and that Tollip knockdown reverses IFNAR1 and IFNGR1 downregulation in SARS-CoV-2 infection, suggesting that Tollip functions as a regulator of Nsp14 innate immune modulation.

## INTRODUCTION

Severe acute respiratory syndrome coronavirus 2 (SARS-CoV-2) has been responsible for over 776 million reported cases and 7.06 million deaths worldwide since 2019 (1). SARS-CoV-2 is an enveloped virus with a positive-sense, single-stranded RNA genome of ∼30 kb (2). The genome encodes 16 non-structural proteins, the structural proteins spike (S), envelope (E), membrane (M), and nucleocapsid (N), and several accessory proteins (3). Among the non-structural proteins is Nsp14, a bifunctional protein with roles in viral RNA synthesis. Nsp14 possesses two distinct domains, one at the N-terminus with exoribonuclease (ExoN) activity that removes nucleotide misincorporations during viral RNA synthesis (4). Nsp14 exonuclease activity is greatly enhanced by the addition of Nsp10, which interacts with the N-terminus of Nsp14, promoting conformational change and enhancing stability (5–7). Due to the high error rate of the coronavirus RNA-dependent-RNA-polymerase (8), the proofreading activity provided by the Nsp10-Nsp14 complex is critical for maintaining the integrity of the large genome (4, 9–11). The second functional domain, found at the Nsp14 C-terminus, is a guanine-N7-methyltransferase (N7-MTase) which contributes to viral RNA cap methylation and virus replication (12–14). This capping promotes translation of the coronavirus mRNA and interferes with innate immune recognition (15, 16).

SARS-CoV-2 modulates innate immunity in a variety of ways. For example, SARS-CoV-2 infection activates NF-κB and MAPK signaling (17–19). Activation of these pathways promotes replication and may contribute to uncontrolled inflammatory responses and cytokine storm that, in some cases, results in acute respiratory distress syndrome, multiorgan failure, and fatal outcomes (20–22). SARS-CoV-2 also antagonizes IFN responses, with a wide variety of mechanisms reported (23). Some of the mechanisms are due to general effects on host cell processes. For example, coronaviruses shut down host protein synthesis through the actions of multiple viral proteins (24–28). In particular, Nsp1 blocks translation by promoting cellular mRNA degradation and by binding to the 40S ribosomal subunit; and it blocks mRNA nuclear export (25, 26, 29–35). ORF6 interacts with importin alpha and nucleoporins, disrupting nuclear-cytoplasmic trafficking (36–42). These activities suppress IFN responses. Other viral proteins may target specific innate immune pathways (23).

Nsp14 modulates innate immunity. Overexpression of Nsp14 causes near-total shutdown of cellular protein synthesis, including synthesis of antiviral IFN-stimulated genes (ISGs) (43). This effect is reversed by mutations that inactivate either the ExoN or the N7-MTase activity and is enhanced by Nsp10 (43). Nsp14 has also been linked to global dysregulation of host transcription, mRNA processing, and nuclear export (44, 45), activities that likely modulate host innate immune responses. Nsp14 has also been implicated in the activation of NF-κB and the suppression of IFNα/β signaling. Activation of NF-κB has been attributed to Nsp14 interaction with inosine-5′-monophosphate dehydrogenase 2; through interaction with the IKK complex (IKKα/ΙΚΚβ/NEMO), which may be recruited due to linear ubiquitination of Nsp14, or by a mechanism that does not involve interaction with the IKK complex (IKKα/ΙΚΚβ/NEMO) (45–51). Mutations designed to abrogate MTase activity impair NF-κB activation, but in one study, N7-MTase inhibitors (nitazoxanide and sinefungin) did not block NF-κB signaling (47). More recently, Nsp14 was described to activate the MAP kinase extracellular-signal-regulated kinase (ERK) and trigger AP-1-dependent gene expression (52). Beyond activation of innate immune pathways, screens to identify SARS-CoV-2 interferon antagonists identified Nsp14 as an inhibitor of IFNβ promoter induction and IRF3 nuclear accumulation (53, 54). Nsp14 antagonizes IFNα/β induced signaling by downregulating the expression of interferon alpha/beta receptor 1 (IFNAR1), one of two proteins that comprise the IFNα/β receptor, possibly through lysosomal degradation (36, 53, 55).

We sought to clarify how Nsp14 modulates diverse innate immune signaling pathways. We demonstrate that Nsp14 not only stimulates NF-κB and ERK but also activates the p38 MAP kinase and Jun amino-terminal kinase (JNK) MAPKs. We further demonstrate that Nsp14 downregulates via a lysosomal pathway not only the IFNAR1 subunit of the IFNα/β receptor but also the IFN-γ receptor 1 (IFNGR1) subunit of the IFNγ receptor. By assessing a panel of Nsp14 point mutants and truncation mutants, we demonstrate that inactivation of N7-MTase disrupts NF-κB and MAPK activation and IFNAR1/IFNGR1 downregulation and that each activity follows the same pattern of sensitivity to the mutants tested. We finally demonstrate that Nsp14 interacts with the host protein Tollip, a multifunctional protein that inhibits NF-κB activation via Toll-like receptor and IL-1R signaling and a acts as a selective autophagy receptor that directs ubiquitinated proteins toward lysosomal degradation (56). Tollip can antagonize Nsp14 activation of NF-κB, and Tollip knockdown counteracts IFNAR1 and IFNGR1 downregulation in the context of SARS-CoV-2 infection.

## RESULTS

### SARS-CoV-2 Nsp14 activates the NF-κB and MAPK signaling pathways and induces cytokine production

**Fig 1:**
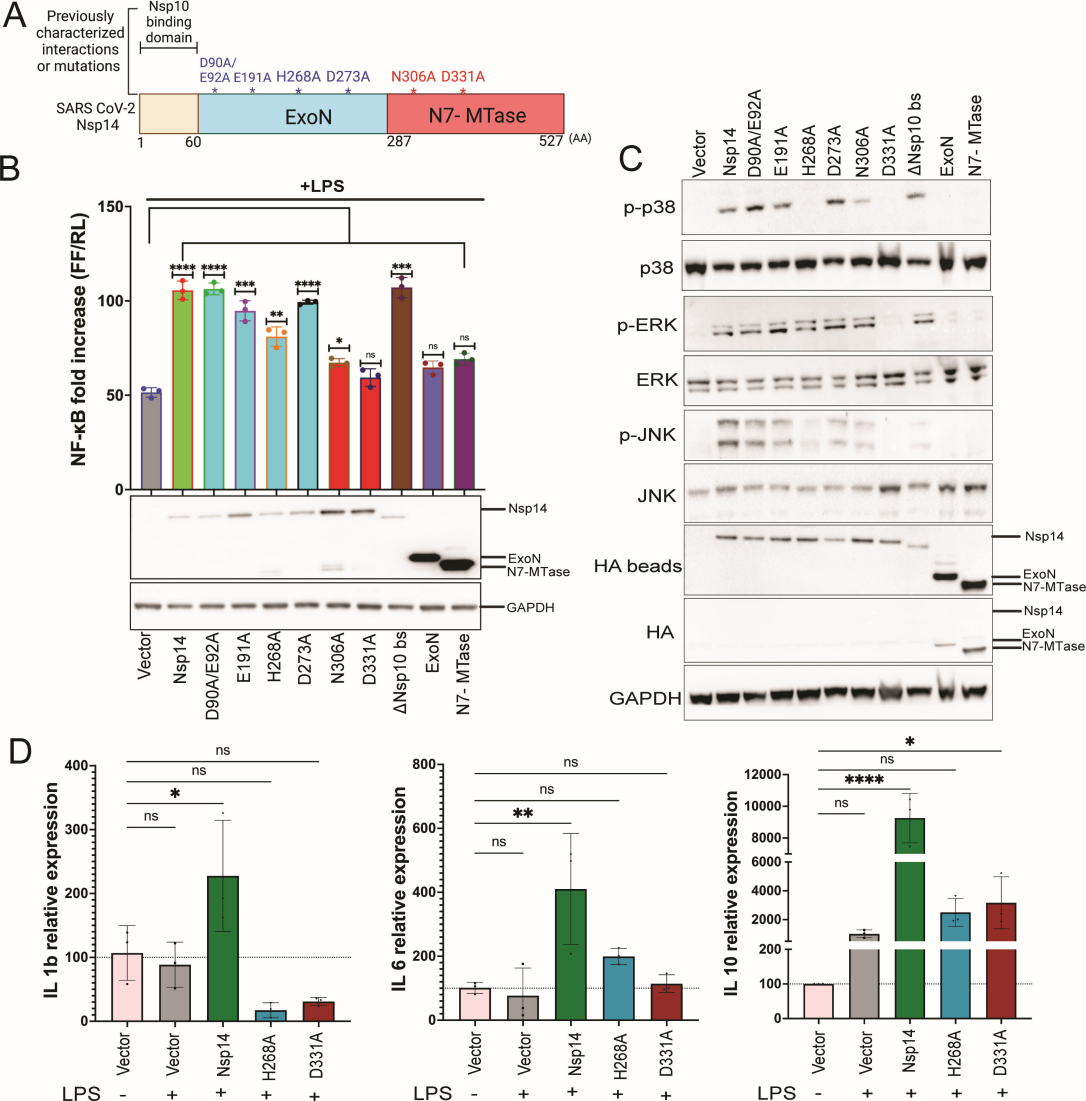
SARS-CoV-2 Nsp14 activates NF-κB and MAPK signaling. (**A**) Schematic depiction of Nsp14 protein domains, including the amino-terminal 60 amino acid residues needed for binding to Nsp10 (tan), the exonuclease (ExoN) domain (blue), and N7-methyltransferase (N7-MTase) domain (red). Amino acid residues at the boundaries of the domains are indicated at the bottom. Key amino acid residues that affect ExoN or N7-MTase activities are indicated at the top. (**B**) HEK293 cells that express TLR4, MD2, and CD14 were transfected with an NF-κB-firefly luciferase (FF) reporter plasmid, a constitutively expressing *Renilla* luciferase (RL), and plasmids that express HA-tagged versions of the indicated proteins. At 24 hours post-transfection, cells were treated with LPS. Eighteen hours later, a dual luciferase reporter assay was performed. The data are presented as fold induction relative to an empty vector, mock-treated control. Error bars represent mean ± SD (*n* = 3). One-way analysis of variance (ANOVA) was used to determine statistical significance (*P ≤ 0.05, **P < 0.01, ***P < 0.001, ****P < 0.0001; ns, not significant). Cell lysates were analyzed by immunoblot. Vector, empty expression plasmid; Nsp14, full-length Nsp14; full-length Nsp14s with point mutations are indicated; ΔNsp10 bs-Nsp14, Nsp14 lacking residues 1–60; ExoN, Nsp14 residues 1–287; N7-MTase, only Nsp14 287–527. (**C**) Immunoblots for total and phosphorylated (p) MAPK pathway proteins in the lysates of HEK293T cells transfected with empty vector or the indicated HA-tagged wild-type and mutant Nsp14s. HA beads, HA-Nsp14 proteins were concentrated by anti-HA immunoprecipitation and then analyzed by immunoblotting. (**D**) Expression levels of IL1b, IL6, and IL10 as determined by quantitative reverse transcription PCR (RT-qPCR) in cells transfected with empty vector (Vector), Nsp14, and the indicated Nsp14 mutants. Cells were mock-treated or treated with LPS, as indicated. Error bars represent mean ± SD (*n* = 3). One-way ANOVA was used to determine statistical significance (*P ≤ 0.05, **P < 0.01, ****P < 0.0001; ns, not significant).

We generated constructs expressing the full-length Nsp14, as well as select point mutants that target exonuclease (ExoN) activity (D90A/E92A, E191A, H268A, D273A) and N7-methyltransferase (N7-MTase) activity (N306A, D331A) (Fig. 1A) (14, 57, 58). We also deleted amino acids 1-60, the Nsp10 binding site (ΔNsp10bs-Nsp14), and generated deletions of either the ExoN or the N7-MTase domains (Fig. 1A) (58). To determine how these Nsp14 expression constructs affect lipopolysaccharide (LPS)-induced activation of NF-κB, reporter gene assays were performed in HEK293T cells stably expressing human TLR4, MD2, and CD14 (hTLR4-MD2-CD14). Full-length, wild-type Nsp14 significantly enhanced LPS induction of the NF-κB reporter, relative to an empty vector, untreated control (Fig. 1B). The ΔNsp10bs-Nsp14 mutant yielded comparable results, indicating that the Nsp10 binding site is dispensable for this activity. Enhancement of NF-κB activity was either similar to wild type or only modestly reduced for point mutations in the ExoN domain. However, constructs containing the N306A or D331A N7-MTase mutations had impaired activity. Impairment was also observed for constructs expressing only the ExoN domain or only the N7-MTase domain, indicating that both domains are required for activity (Fig. 1B). Expression of the Nsp14 constructs was confirmed by immunoblot, with some variation in levels.

Because SARS-CoV-2 activates pro-inflammatory MAPK signaling (18, 19, 51), we investigated the capacity of Nsp14 to activate the MAPK pathways. HEK293T cells were transfected with the wild type and mutant Nsp14s, and the status of MAPK family proteins in lysates was assessed by immunoblot. Because HA-Nsp14 expression was difficult to detect, anti-HA immunoprecipitations were performed, and the precipitated material was assessed by immunoblotting. Total levels of p38, ERK, and JNK were largely unaffected by Nsp14. However, Nsp14 increased phosphorylation of the three major families of MAPKs as evidenced by p38, ERK, and JNK phosphorylation (Fig. 1C). For ERK and JNK, activation was also observed for the ΔNsp10 bs mutant and for the ExoN D90A/E92A, E191A, H268A (weak for JNK), and D273A point mutants. For the N7-MTase mutants, some degree of activation was seen for the N306A mutant, while the D331A mutant did not stimulate the phosphorylation of ERK or JNK. The two mutants may behave differently because N306A retains ~30% residual activity in an *in vitro* assay of N7-MTase activity, while this activity was nearly abolished for D331A (14). The findings were very similar for p38, with induction of phosphorylation detected for most mutants but not for the N7-MTase D331A mutant. For p38, the ExoN H268A mutant also failed to induce phosphorylation, although the remaining three point mutants in ExoN retained the ability to promote phosphorylation. The separated ExoN or N7-MTase domains failed to activate p38, ERK, or JNK. Overall, the results indicate that Nsp14 activates both the NF-κB and MAPK signaling pathways, that neither of its major enzymatic domains, when individually expressed, is sufficient for the activation, and that the N7-MTase domain appears to play a critical role based on the loss of activation by the catalytically dead D331A mutant.

Because the NF-κB and MAPK signaling pathways contribute to inflammatory responses, we determined the impact of Nsp14 expression on cytokine induction in HEK293-hTLR4-MD2-CD14 cells in the presence of LPS. We included wild-type Nsp14 and mutants H268A and D331A, which exhibited impaired activation of NF-κB and MAPK. Wild-type Nsp14 enhanced expression of the typically proinflammatory cytokines IL-1β and IL-6 and the anti-inflammatory cytokine IL-10 (Fig. 1D). The induction of IL-1β, IL-6, and IL-10 was reduced for the mutants tested, correlating with the NF-κB and MAPK pathway assays (Fig. 1B and C). Similar data were obtained for the chemokines IP-10 (CXCL10), MIP-1β (CCL4), and RANTES (CCL5) (Fig. S1). The extent to which NF-κB and MAPK signaling contributes to the activation of each cytokine was not further addressed.

### SARS-CoV-2 Nsp14 downregulates expression of IFNAR1 and IFNGR1, impairing interferon signaling pathways

**Fig 2:**
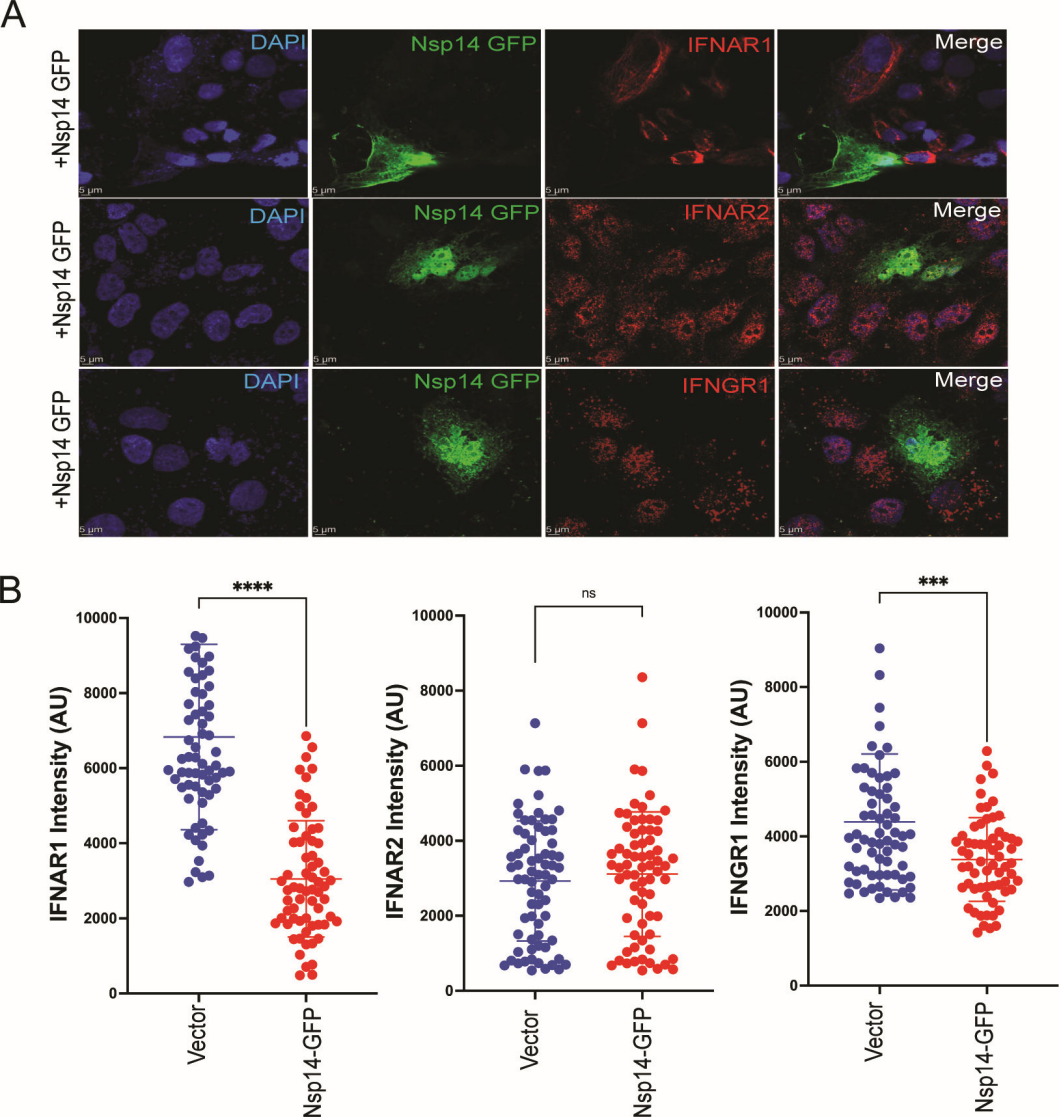
SARS-CoV-2 Nsp14 downregulates the expression of IFNAR1 and IFNGR1. (**A**) Confocal laser scanning microscopy image of interferon receptor expression levels in Huh7 cells transfected with an Nsp14-GFP expression plasmid. Blue, DAPI staining of nuclei. Green, Nsp14-GFP. Red, the indicated endogenous receptor. (**B**) Quantification of IFNAR1, IFNAR2, and IFNGR1 staining intensity in cells transfected with empty vector (Vector) or Nsp14-GFP. Each dot indicates the value for a single cell. Error bars represent mean ± SD (*n* = 3). Unpaired two-tailed Student’s *t* test was used to determine statistical significance (***P < 0.001, ****P < 0.0001; ns, not significant).

We examined the expression of endogenous interferon receptors in cells transfected with an Nsp14-GFP fusion construct. Cells expressing Nsp14-GFP exhibited very low or negligible levels of both IFNAR1 and IFNGR1 (Fig. 2A). Levels of IFN alpha receptor 2 (IFNAR2), in contrast, were unaffected by Nsp14-GFP (Fig. 2A). When quantified, IFNAR1 and IFNGR1 staining intensity was significantly lower for cells expressing Nsp14-GFP than for cells not expressing Nsp14-GFP, while IFNAR2 staining intensity did not differ between Nsp14-GFP-positive and -negative cells (Fig. 2B). Cultures not transfected with Nsp14-GFP displayed relatively uniform expression of all three receptors (Fig. S2).

**Fig 3:**
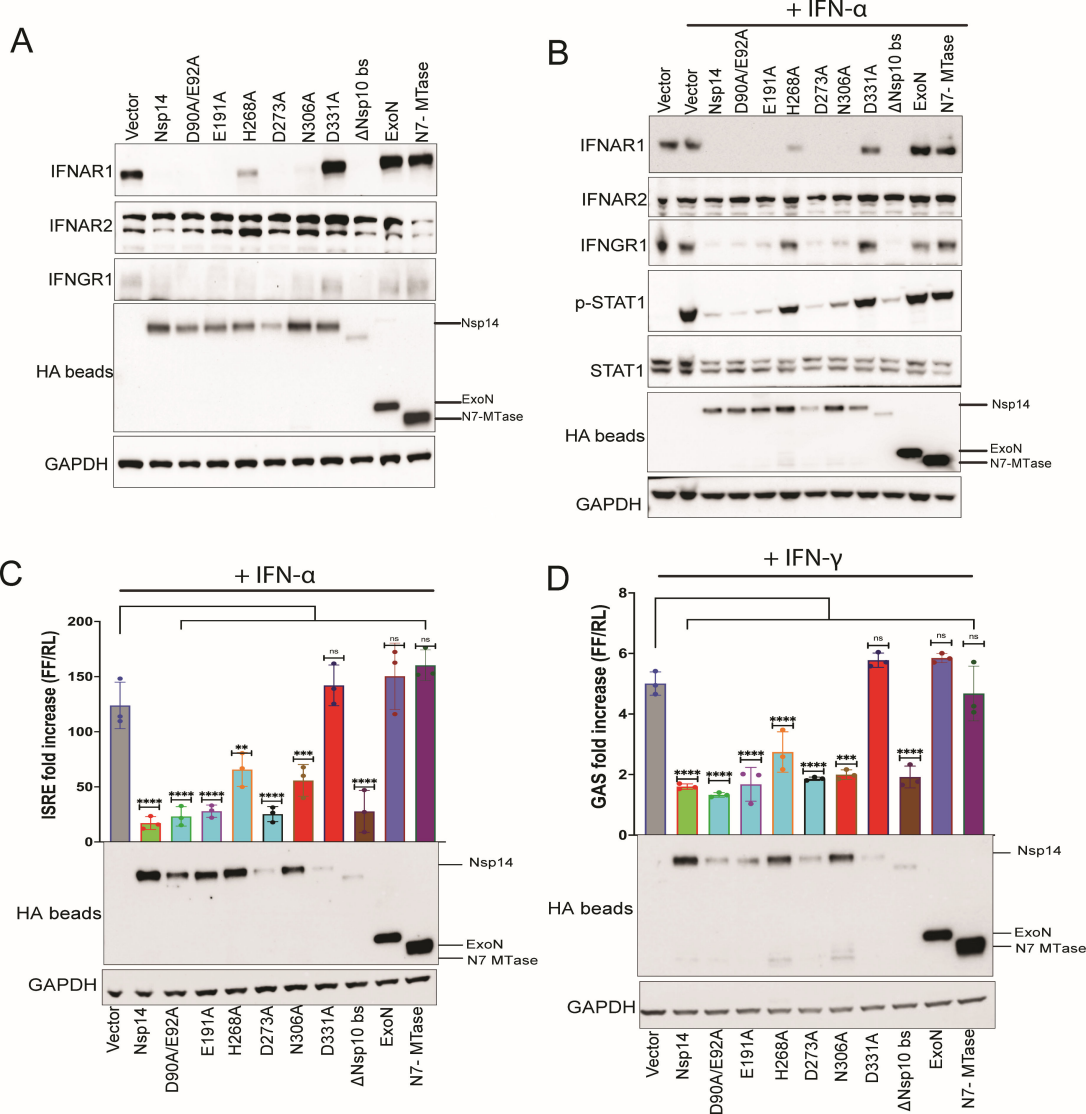
Nsp14 inhibits IFNα and IFNγ signaling via downregulation of IFNAR1 and IFNGR1. (**A**) Immunoblots assessing levels of endogenous IFNAR1, IFNAR2, IFNGR1, and glyceraldehyde-3-phosphate dehydrogenase (GAPDH) in HEK293T cells transfected with empty vector (Vector) or plasmids expressing the indicated HA-tagged Nsp14s. HA-Nsp14 proteins were concentrated by immunoprecipitation with beads conjugated to anti-HA antibodies (indicated by the label HA beads) and then analyzed. (**B**) Immunoblots to assess the effect of Nsp14 on STAT1 phosphorylation 30 minutes after IFNα addition. Transfections were performed as in panel A. Blotting included antibodies to detect total and tyrosine-phosphorylated STAT1. (**C**) Effects of transfecting the indicated expression plasmids on transcriptional responses to IFNα as assessed by reporter gene assay. HEK293T cells were transfected with an interferon-stimulated response element (ISRE)-firefly luciferase reporter plasmid, a constitutively expressing *Renilla* luciferase, and the indicated plasmids. At 24 hours post-transfection, cells were treated with IFNα. Twenty-four hours later, luciferase activities were determined. Firefly luciferase values were normalized to *Renilla* luciferase values, and data are reported as fold increase relative to empty vector, mock-treated samples. Error bars represent mean ± SD (*n* = 3). One-way ANOVA was used to determine statistical significance (**P < 0.01, ***P < 0.001, ****P < 0.0001; ns, not significant). Cell lysates were analyzed by immunoblot. (**D**) Effects of transfecting the indicated expression plasmids on transcriptional responses to IFNγ as assessed by reporter gene assay. Transfections were performed as in panel C, except a gamma-activated sequence (GAS)-firefly luciferase reporter plasmid was used in place of the ISRE firefly luciferase plasmid, and cells were treated with 500 U/mL IFN-γ. Analysis was as in panel C. Cell lysates were analyzed by immunoblot. Labeling of immunoblots for panels C and D was the same as for panel A.

We next assessed the impact of our panel of Nsp14 constructs on IFNAR1, IFNAR2, and IFNGR1 by immunoblot. Wild-type Nsp14 substantially reduced levels of endogenous IFNAR1 while levels of IFNAR2 were unaffected. Although the detection of IFNGR1 was relatively inefficient in this experiment, its levels were also reduced by wild-type Nsp14 (Fig. 3A). Results were similar for the Nsp14 mutants tested, with the exception of the D331A mutant and the individually expressed ExoN and N7-MTase domains, which failed to reduce levels of IFNAR1 or IFNGR1. The H268A mutant exhibited an intermediate phenotype. These results mirror our NF-κB and MAPK signaling pathway findings, where both domains of the bifunctional Nsp14 protein were required to observe the phenotypes, and where the D331A point mutant, which abolishes N7-MTase activity, resulted in a complete loss of activity.

To investigate the effects of IFN receptor dysregulation on downstream signaling, we examined STAT1, a key transcription factor that mediates IFNα/β and IFNγ signaling, in the absence and presence of IFN in cells expressing vector alone or the Nsp14 constructs. Thirty minutes post-IFN addition, basal STAT1 levels were similar for all proteins tested (Fig. 3B). As expected, IFN treatment resulted in the appearance of tyrosine phosphorylated STAT1 (p-STAT1) in the vector control. Consistent with the observed downregulation of IFNAR1, levels of p-STAT1 were diminished in the presence of wild-type Nsp14 (Fig. 3B). Similar reductions were observed for the Nsp14 mutants, with the exceptions of H268A, D331A, and the individual ExoN and N7-MTase domains, all of which displayed activation similar to controls. As was the case in the absence of IFN (Fig. 3A), both IFNAR1 and IFNGR1 levels were substantially reduced in the presence of IFN treatment for cells expressing wild-type Nsp14 (Fig. 3B). This was also true for the Nsp14 mutants, again with the exception of H268A, D331A, and the individual ExoN and N7-MTase domains. Finally, as seen in the absence of IFN treatment, IFNAR2 levels were unaffected by Nsp14 in the presence of IFN, indicating a specificity to dysregulation of receptor levels (Fig. 3B).

When interferon-stimulated response element (ISRE)-firefly luciferase reporter gene assays were performed in the absence or presence of wild-type or mutant Nsp14s, wild-type Nsp14 repressed the activation of the ISRE reporter in the presence of IFNα (Fig. 3C). This repression was relieved for the D331A mutant and for the ExoN and N7-MTase individual domain mutants (Fig. 3C). Similar findings were obtained using cells transfected with an interferon gamma-activated sequence (GAS)-firefly luciferase reporter plasmid and treated with IFNγ (Fig. 3D). Expression of each Nsp14 construct was confirmed by immunoblot analysis for these studies (Fig. 3C and D).

### Nsp14 downregulation of IFNAR1 and IFNGR1 involves a lysosomal pathway

**Fig 4:**
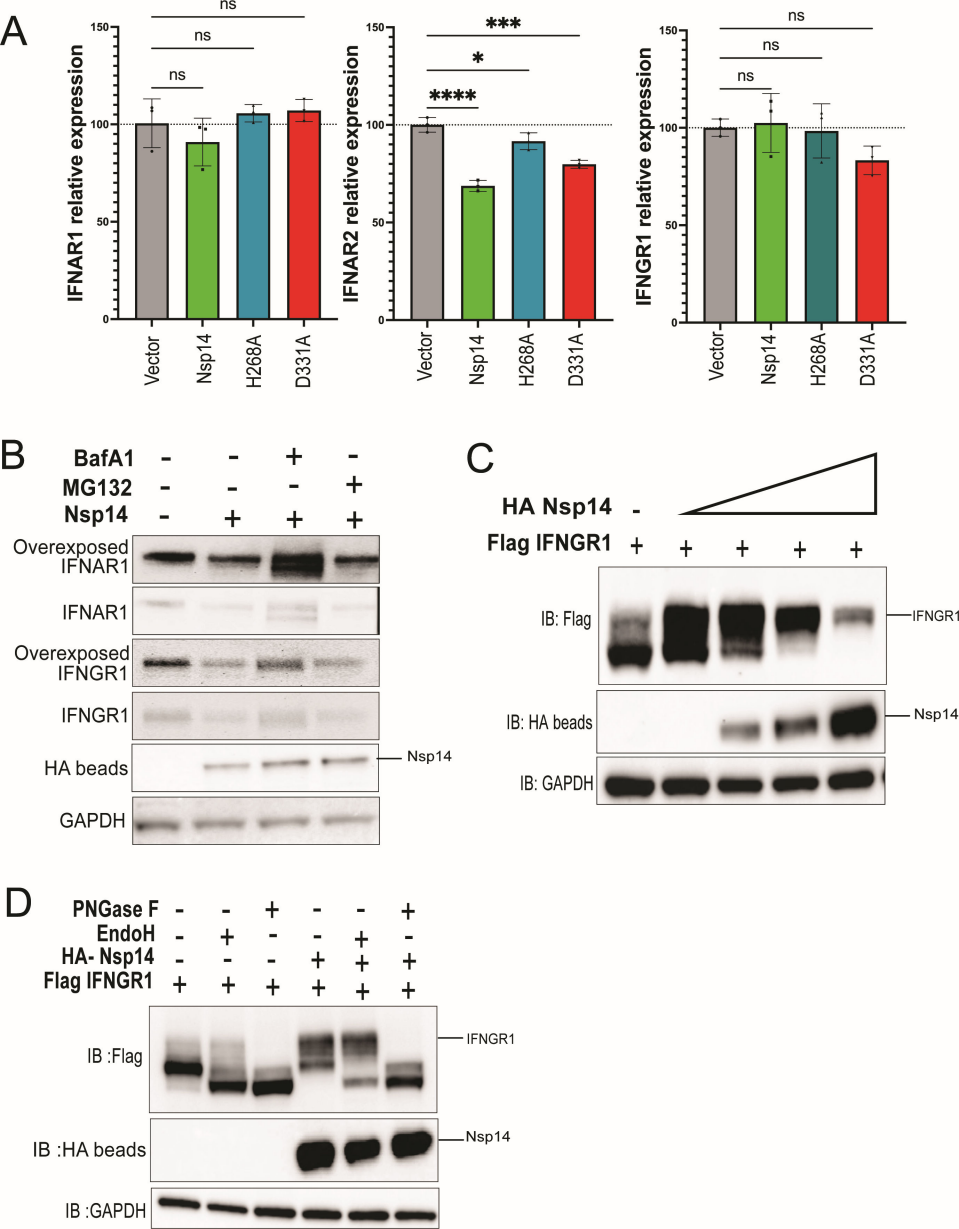
Mechanisms of IFNAR1 and IFNGR1 downregulation. (**A**) RT-qPCR analysis of IFNAR1, IFNAR2, and IFNGR1 mRNA expression levels in Huh7 cells 24 hours post-transfection with empty vector (Vector), Nsp14, and the indicated Nsp14 mutants. Error bars represent mean ± SD (*n* = 3). One-way ANOVA was used to determine statistical significance (*P ≤ 0.05, ***P < 0.001, ****P < 0.0001; ns, not significant). (B) Immunoblot for endogenous level of IFNAR1 and IFNGR1 in HEK293T cells overexpressing HA-Nsp14 in the absence or presence of BafA1 (2 µM) or MG132 (50 µM) for 6 hours before harvesting. HA bead samples were concentrated by immunoprecipitation with anti-HA antibody-conjugated beads prior to immunoblotting. GAPDH served as a loading control. (**C**) Immunoblot demonstrating effects of increasing concentrations of Nsp14 on overexpressed IFNGR1 levels and migration on SDS-PAGE. HA beads, samples were concentrated by immunoprecipitation with anti-HA antibody-conjugated beads prior to immunoblotting. GAPDH served as a loading control. (**D**) Immunoblot of cell lysates from HEK293T overexpressing Flag-IFNGR1 and HA-Nsp14 that were mock-treated or treated with PNGase F or Endo H. GAPDH served as a loading control. HA bead samples were concentrated by immunoprecipitation with anti-HA antibody-conjugated beads prior to immunoblotting.

Expression of Nsp14 induces widespread transcriptional changes resembling those following SARS-CoV-2 infection, and these effects require the N7-MTase domain (45). SARS-CoV-2 Nsp14 also inhibits nuclear mRNA processing and export (44). To investigate whether Nsp14 modulates interferon receptor expression at the mRNA level, IFNAR1, IFNAR2, and IFNGR1 transcript levels were quantified by quantitative reverse transcription PCR (RT-qPCR). IFNAR1 and IFNGR1 mRNA levels were similar for cells expressing wild-type Nsp14, H268A or D331A, as compared with the vector control (Fig. 4A). Despite not exhibiting obvious differences at the protein level, IFNAR2 transcript levels were modestly reduced to varying degrees with expression of wild-type Nsp14 or the D331A or H268A mutants (Fig. 4A). Therefore, Nsp14 downregulation of IFNAR1 and IFNGR1 expression occurs at a post-transcriptional level. A prior study found that Nsp14 inhibition of translation was abrogated by mutations that disrupt ExoN or N7-MTase activities (43). Because the ExoN mutants retain downregulation activity, translation inhibition does not explain decreases in IFNAR1 and IFNGR1 levels.

To further address the mechanism of downregulation, we inhibited the proteasomal and lysosomal pathways. Reduction of IFNAR1 and IFNGR1 was maintained in the presence of the proteasome inhibitor MG132, but was diminished in the presence of the lysosomal inhibitor bafilomycin A1 (BafA1) (Fig. 4B). Increasing HA-Nsp14 resulted in a dose-dependent reduction in the expression of co-transfected Flag-IFNGR1 (Fig. 4C). On immunoblot, at least two bands were observed for over-expressed IFNGR1, with the faster-migrating band most affected by Nsp14 (Fig. 4C). To determine whether the multiple bands might represent differentially glycosylated forms, the lysates were treated with peptide:N-glycosidase F (PNGase F), which removes almost all types of *N*-linked (Asn-linked) glycosylation, and with endoglycosidase H (Endo H), which selectively removes high mannose and certain hybrid types of *N*-linked carbohydrates. In the absence of Nsp14, Flag-IFNGR1 was susceptible to digestion by both PNGase F and Endo H, with a shift to the faster-migrating form (Fig. 4D). With co-expression of Nsp14, Flag-IFNGR1 remained susceptible to digestion by PNGase F but was substantially resistant to Endo H (Fig. 4D). This suggests that Nsp14 selectively promotes degradation of Flag-IFNGR1 forms in the ER and early regions of the Golgi complex, allowing more Endo H-resistant forms to predominate.

### Identification of the autophagy selective receptor protein Tollip as an interacting partner of SARS-CoV-2 Nsp14

**Fig 5:**
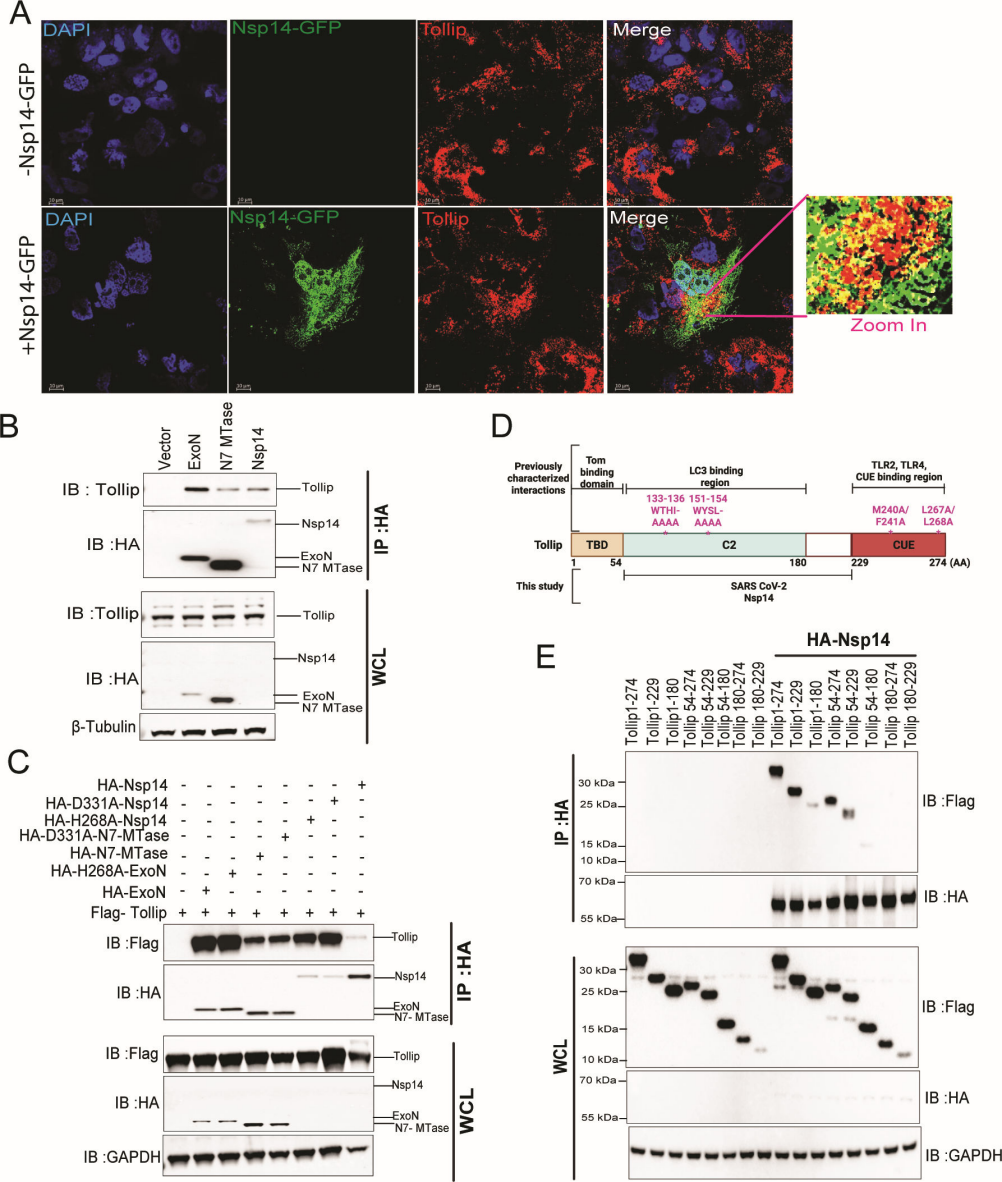
SARS-CoV-2 Nsp14 co-localizes and interacts with Tollip. (**A**) Immunofluorescence to assess localization of Nsp14-GFP and endogenous Tollip in Huh7 cells. Blue, DAPI (nuclei); green, Nsp14-GFP; red, Tollip. (**B**) Co-IP of endogenous Tollip with transfected empty vector (Vector), HA-Nsp14 ExoN domain, N7 MTase domain, or full-length Nsp14. Immunoblots of the immunoprecipitations (IP:HA) and whole-cell lysates (WCL) are shown. Blots were probed with anti-Tollip, anti-HA, and anti-β-tubulin antibodies, as indicated. (**C**) Interaction of Flag-Tollip and the indicated HA-tagged Nsp14 constructs was assessed by coIP. IP:HA, samples subjected to anti-HA immunoprecipitation. Immunoblots of the immunoprecipitations (IP:HA) and WCL are shown. Blots were probed with anti-Flag, anti-HA, and anti-GAPDH antibodies, as indicated. (**D**) Schematic depiction of Tollip protein domains and key residues for catalytic activity and ubiquitin binding. TBD, Tom1 binding domain; C2, central conserved 2 (C2) domain involved in Ca^2+^-dependent membrane-targeting, LC3 binding; CUE, coupling of ubiquitin to ER degradation domain. Amino acid residues at the boundaries of domains are indicated below the diagram. Point mutants used in this study are indicated above the diagram. The region that interacts with Nsp14, as determined by this study, is indicated below the diagram. (**E**) Domain mapping studies. HEK293T cells were co-transfected with empty vector or HA-Nsp14 and the indicated Flag-tagged Tollip constructs, and anti-HA immunoprecipitations were performed. Immunoblots of the immunoprecipitations (IP:HA) and WCL are shown. Blots were probed with anti-Flag, anti-HA, and anti-GAPDH antibodies, as indicated.

Tollip is a ubiquitously expressed adaptor protein that plays roles in diverse intracellular signaling pathways. Tollip delivers cargo proteins to the lysosome for degradation, including the receptors for IL-1 and TGFβ (59, 60). Tollip has also been implicated in the selective clearance of aberrant ER cargos (61). Ongoing work identified an interaction between Nsp14 and Tollip. This is illustrated by immunofluorescence analysis in which Nsp14 colocalizes with endogenous Tollip (Fig. 5A). Coimmunoprecipitation analysis also demonstrated interaction between endogenous Tollip and HA-tagged wild-type Nsp14 and with the individual ExoN and N7-MTase domains (Fig. 5B).

Because Tollip interacts with both the Nsp14 ExoN and MTase domains, we tested whether the catalytic activities of the individual domains were required for the interaction. Flag-tagged Tollip immunoprecipitated with HA-tagged wild-type NSP14, with the exonuclease mutant (H268A) and the N7-MTase mutant (D331A), whether as individual domains or in the context of the full-length Nsp14 (Fig. 5C). Therefore, Nsp14 catalytic activities are dispensable for the Tollip interaction.

Tollip is modular, with an N-terminal Tom1-binding domain (TBD) involved in endosomal sorting, a central conserved 2 (C2) domain which interacts with phosphoinositides and LC3, and a C-terminal CUE domain involved in ubiquitin-binding (Fig. 5D) (56, 62, 63). To map interaction domains, we generated a series of truncation mutants. Flag-tagged Tollip constructs were co-expressed with HA-tagged Nsp14. When HA-Nsp14 was immunoprecipitated, the Tollip domain spanning amino acids 54–229 or constructs in which this region was retained were co-precipitated (Fig. 5E). The Tollip 1-180 and 54-180 constructs co-precipitated very weakly, suggesting that 54–180, which corresponds to the C2 domain, may be sufficient for interaction. However, because 54-229 clearly binds better, residues 180–229 contribute to a strong interaction.

### Tollip partially reverses Nsp14-mediated NF-κB activation and cytokine expression

**Fig 6:**
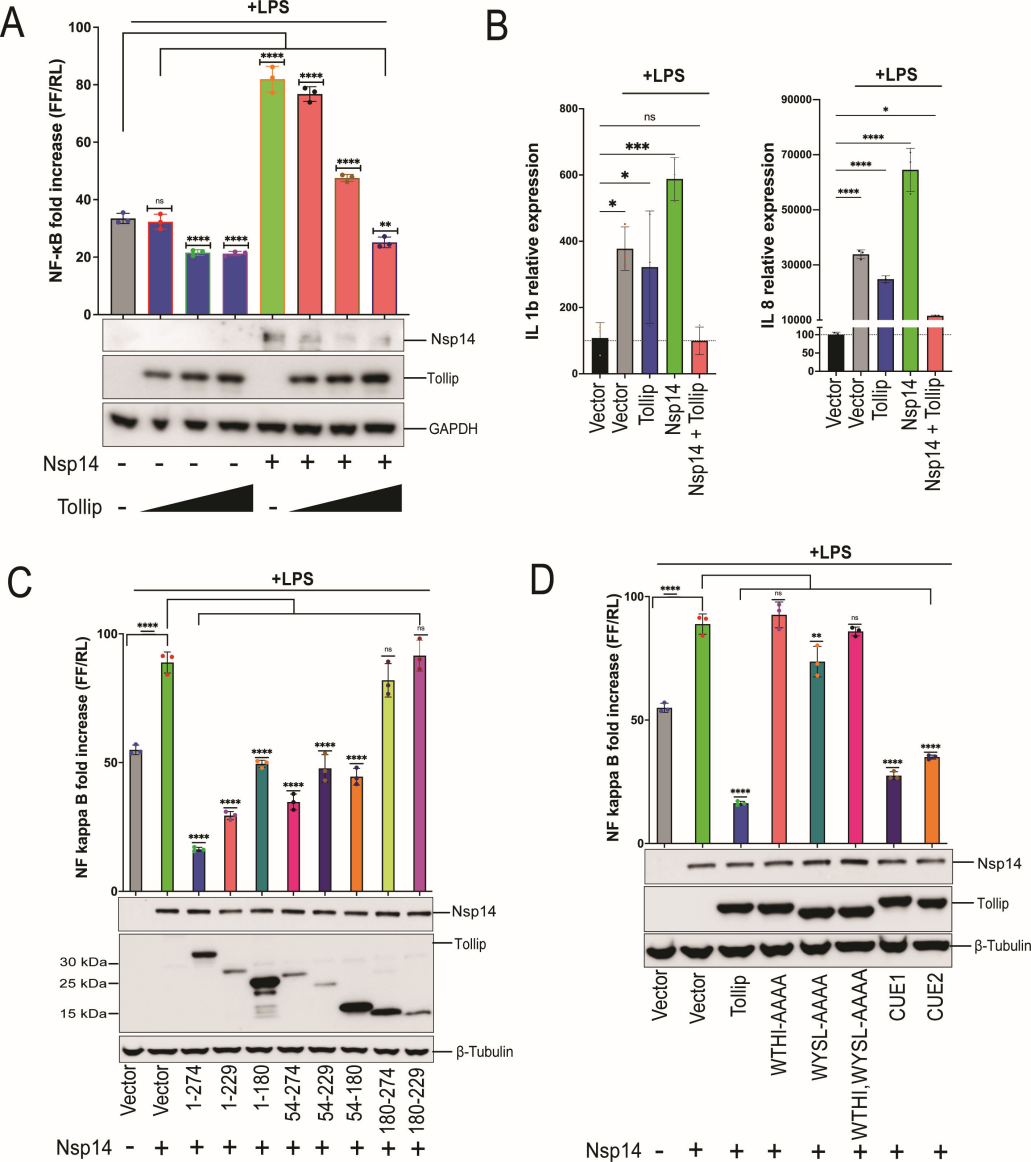
Tollip counteracts Nsp14-mediated NF-κB activation. (**A**) Effect of Tollip levels on Nsp14 activation of NF-κB. HEK293 cells that express TLR4, MD2, and CD14 were transfected with an NF-κB-firefly luciferase (FF) reporter plasmid, a constitutively expressing *Renilla* luciferase (RL), HA-Nsp14, and increasing amounts of Flag-tagged Tollip. Eighteen hours later, a dual luciferase reporter assay was performed. Firefly luciferase activity was normalized to *Renilla* luciferase activity. The data are presented as fold induction relative to an empty vector, mock-treated control. Error bars represent mean ± SD (*n* = 3). One-way ANOVA was used to determine statistical significance (**P < 0.01, ****P < 0.0001; ns, not significant). Cell lysates were analyzed by immunoblot for the indicated proteins. (**B**) IL1β and IL8 mRNA levels in HEK293 cells that express TLR4, MD2, and CD14, transfected with empty vector (Vector), Tollip, and/or Nsp14 plasmids, in the absence or presence of LPS, as indicated. Error bars represent mean ± SD (*n* = 3). One-way ANOVA was used to determine statistical significance (*P ≤ 0.05, ***P < 0.001, ****P < 0.0001; ns, not significant). (**C**) Domain mapping studies. HEK293-hTLR4-MD2-CD14 cells were transfected with an NF-κB-firefly luciferase reporter plasmid and the indicated expression plasmids. The absence or presence of Nsp14 expression plasmids is indicated at the bottom. At 24 hours post-transfection, cells were treated with LPS, and a dual luciferase reporter assay was performed the following day. Firefly luciferase activity was normalized to *Renilla* luciferase activity. Error bars represent mean ± SD (*n* = 3). One-way ANOVA was used to determine statistical significance (****P < 0.0001; ns, not significant). Cell lysates were analyzed by western blot. (**D**) Tollip point mutant studies. An NF-κB-firefly luciferase reporter assay was performed as above. The indicated protein expression plasmids were assayed. Error bars represent mean ± SD (*n* = 3). One-way ANOVA was used to determine statistical significance (**P < 0.01, ****P < 0.0001; ns, not significant). Cell lysates were analyzed by western blot.

Tollip regulates inflammatory pathways and inhibits NF-κB signaling triggered by IL-1β, Toll-like receptor (TLR)2, and TLR4 (64–66). We therefore asked whether Tollip expression would affect the Nsp14 activation of NF-κB. As above (Fig. 1B), expression of Nsp14 significantly increased LPS-stimulated NF-κB reporter activity; this was dampened, in a dose-dependent manner, by overexpressed Tollip (Fig. 6A). Expression of Nsp14 also increased the LPS-induced expression of IL-1β, IL-8 (Fig. 6B), IP-10, MIP-1β, and RANTES transcripts (Fig. S3), but co-expression of Tollip reduced these effects (Fig. 6B; Fig. S3).

We mapped this inhibitory activity with Tollip truncation mutants. As before, full-length Tollip inhibited Nsp14 enhancement of NF-κB reporter activity (Fig. 6C). The Tollip 1-229 mutant, which contains the TBD and the C2 domain but lacks the CUE domain, retained substantial inhibitory activity (Fig. 6C). This was also true for the 54-274 mutant that lacks the TBD but contains both the C2 and CUE domains (Fig. 6C). Mutants 180-274 and 180-229, both of which lack the C2 domain, displayed little activity (Fig. 6C). Mutant 1-180, which contains the C2 domain but not additional C-terminal sequences, as well as mutants 54-229 and 54-180 showed intermediate inhibitory activity (Fig. 6C). These results support the importance of the C2 domain and C-terminal sequences adjacent to it and indicate that multiple regions of the protein may be required for full activity, as none of the truncation mutants are as active as full-length Tollip. We also examined the Tollip deletion mutants in the presence of LPS but in the absence of Nsp14. Overall, a similar pattern of inhibitory activities was noted, with the 1-229 and 54-274 mutants showing the greatest activity, although the 1-180 mutant was inactive in this context (Fig. S4).

We also evaluated Tollip mutants with impaired LC3 and ubiquitin binding. We generated Tollip alanine substitution mutations (i) in the two LC3-interacting motifs of the C2 domain, both separately and combined: W133A, T134A, H135A, I136A (WTHI-AAAA); W151A, Y152A, S153A, L154A (WYSL-AAAA); and WTHI-AAAA/WYSL-AAAA and (ii) in the two ubiquitin-binding motifs of the CUE domain: M240A/F241A (CUE1) and L267A/L268A (CUE2) (Fig. 6D) (67, 68). The LC3 binding mutants did not inhibit Nsp14-induced NF-κB reporter activity, while the ubiquitin binding mutants still inhibited (Fig. 6D), indicating that association with LC3 but not with ubiquitin may be important for Tollip’s antagonism of Nsp14. In the absence of Nsp14, a similar pattern was observed, although only partial loss of activity was seen with two of the LC3 binding mutants (Fig. S4B).

### IFNAR1 and IFNGR1 levels are reduced in the context of SARS-CoV-2 infection, and this is blunted when Tollip expression is silenced

**Fig 7:**
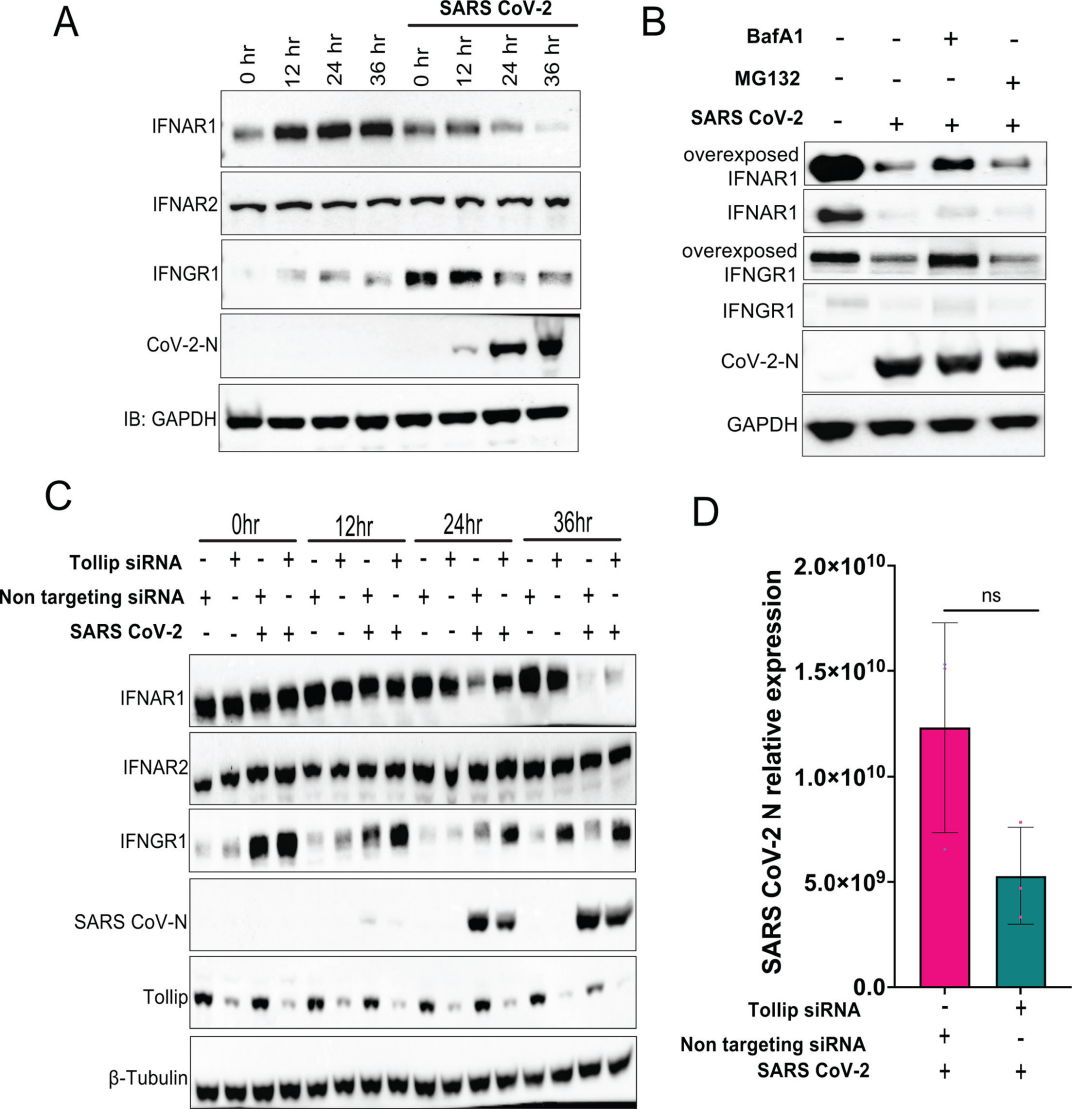
Downregulation of interferon receptor expression during SARS-CoV-2 infection with and without Tollip knockdown. (**A**) Effects of SARS-CoV-2 infection on endogenous IFNAR1, IFNAR2, and IFNGR1 levels. A549-ACE2 cells were mock-infected or infected with SARS-CoV-2 (MOI = 1). Lysates for immunoblotting were prepared at the indicated time points. Blots were probed with antibodies for IFNAR1, IFNAR2, IFNGR1, SARS-CoV-2 N, and GAPDH, as indicated. (**B**) Immunoblot for endogenous IFNAR1 and IFNGR1 following mock-infection or SARS-CoV-2 infection for 18 hours at MOI = 1. As indicated, infected cells were treated with BafA1 (2 µM) or MG132 (50 µM) for 6 hours before harvesting. GAPDH was included as a loading control. (**C**) Immunoblot analysis of A549-ACE2 cells transfected with non-targeting or Tollip-specific siRNA and uninfected or infected with SARS-CoV-2 (MOI = 1) for the indicated times post-infection. Blots were probed with antibodies for IFNAR1, IFNAR2, IFNGR1, SARS-CoV-2 N, and β-tubulin, as indicated. (**D**) RT-qPCR analysis of SARS-CoV-2 N mRNA expression level in A549-ACE2 transfected with non-targeting or Tollip-specific siRNA and infected with SARS-CoV-2 (MOI = 1). N mRNA levels were normalized to β-actin mRNA levels. Error bars represent mean ± SD (*n* = 3).

To address the effects of SARS-CoV-2 infection on IFNAR1 and IFNGR1 and the potential role for Tollip, we examined endogenous levels of interferon receptors in A549 cells transduced with angiotensin-converting enzyme 2 (A549-ACE2), in the absence and presence of SARS-CoV-2 infection at a multiplicity of one. Without infection, IFNAR1 levels were stable. After infection, viral N protein levels increased, as expected. A reduction in IFNAR1 levels was apparent at 24 hours and levels decreased further at 36 hours post-infection (Fig. 7A). Levels of IFNGR1 were also diminished at 24 and 36 hours after infection when compared with the 0 hour time point (Fig. 7A). In this case, for unclear reasons, the initial time point showed higher levels of IFNGR1 in the context of infection as compared with uninfected control cells. IFNAR2 levels remained essentially constant throughout the infection and were similar to the uninfected controls. Furthermore, GAPDH levels were similar at all time points. This decrease of interferon receptor levels in the context of SARS-CoV-2 infection mirrors our findings following Nsp14 transfection (Fig. 3A and B). Inhibition of lysosomal degradation by BafA1, which was added to the cells for 6 hours beginning 18 hours post-infection, partially rescued levels of IFNAR1 and fully rescued levels of IFNGR1 without significantly impacting viral N levels (Fig. 7B). This points to lysosomal activity as contributing to receptor downregulation. In contrast, treatment with the proteasome inhibitor MG132 had no effect. This also agrees with our findings in cells transfected with Nsp14 (Fig. 4B).

To evaluate the role of Tollip in interferon receptor downregulation mediated by SARS-CoV-2, we employed siRNA‐mediated knockdown of Tollip, followed by infection. A non-targeting negative control siRNA was included as a control. Depletion of Tollip impaired IFNAR1 and IFNGR1 downregulation, most evidently at 24 and 36 hours post-infection (Fig. 7C). At the later time point, when Tollip levels were reduced, levels of IFNAR1 and IFNGR1 increased relative to the non-targeting control but remained lower than at the time of infection. IFNAR2 levels were not significantly impacted by Tollip depletion. A modest reduction in SARS-CoV-2 N protein levels was detected by immunoblot at 24 hours post-infection in the setting of Tollip knockdown (Fig. 7C). Therefore, we performed RT-qPCR to examine levels of the N gene-containing RNAs at 24 hours post-infection and observed only a modest decrease in the presence of Tollip knockdown as compared with the control, and the difference did not reach statistical significance (Fig. 7D). Overall, these data suggest a role for Tollip in downregulation of IFNAR1 and IFNGR1 in the context of SARS-CoV-2 infection.

## DISCUSSION

Our data demonstrate that full-length Nsp14 and a mutant lacking the N-terminal Nsp10 binding domain are able to stimulate NF-κB responses to LPS and activate all three of the main MAPK families (69). Likewise, point mutants previously reported to disrupt Nsp14 ExoN activity largely retained the capacity to augment NF-κB and MAPK activation. The one exception was the H268A mutant. This mutant was modestly less potent for NF-κB activation. It promoted ERK phosphorylation and exhibited only modest, albeit detectable, JNK phosphorylation, but p38 MAPK phosphorylation was not detected. Regardless, the data with the other ExoN mutants suggest that Nsp14 exonuclease activity is not required for MAPK activation. In contrast, mutations that impair N7-MTase activity lost NF-κB and MAPK-enhancing activity, suggesting that this enzymatic function may be needed for stimulation of these pathways. Expression of either of the individual ExoN or MTase domains was also insufficient to stimulate NF-κB or MAPK. The latter observations could be an additional reflection of the need for N7-MTase activity. That is, for SARS-CoV Nsp14, the ExoN domain, although not ExoN enzymatic activity, was found to be required for N7-MTase activity (70). Consistent with our data, other studies demonstrated that NF-κB activation was abrogated by mutations that disrupt Nsp14 methyltransferase activity but not by mutants that prevent exonuclease activity (45, 47, 48). Furthermore, expression of the ExoN or MTase domains was not sufficient to activate NF-κB (46, 48, 50).

While our manuscript was in preparation, a publication reported Nsp14 activation specifically of ERK and triggering of AP-1-dependent gene expression (52). In contrast with our findings, this study reported a lack of Nsp14 activation of p38 and JNK phosphorylation. This could be explained by the relatively high basal levels of phospho-p38 and phospho-JNK in the assays of Li et al. (52). Another notable difference was the capacity of the ExoN domain, expressed alone, to trigger ERK phosphorylation (52). Why this was seen in one study but not ours is unclear, but the two studies used different cell lines (HEK293T versus A549). Future studies should assess the function of Nsp14 in primary human cells.

How SARS-CoV-2 engages with and evades IFN responses has been a subject of intense interest (23, 71–74). Nsp14 has been implicated in the inhibition of several aspects of the IFN response (53, 54). Nsp14 was reported to antagonize type I IFN signaling and, on immunoblot, to downregulate expression of the IFNAR1 chain of the IFNα receptor (36, 53, 55). Our data confirm that Nsp14 can block type I IFN signaling through downregulation of IFNAR1. We also demonstrated no effect on IFNAR2. This downregulation is sufficient to block IFNα-induced STAT1 phosphorylation and ISG upregulation. We also demonstrated that Nsp14 downregulates IFNGR1. Treatment of cells with BafA1, an inhibitor of autophagosome-lysosome fusion and lysosome acidification (75), partially rescues both IFNAR1 and IFNGR1 from downregulation by Nsp14. This is consistent with an earlier study that suggested Nsp14 decreases levels of IFNAR1 by promoting degradation via the lysosome (55) and extends the observation to IFNGR1. Interestingly, our studies with Endo H and PNGase F digestion suggest that Endo H-sensitive, less mature forms of IFNGR1 are preferentially targeted by Nsp14, at least when IFNGR1 is over-expressed. These are likely forms present in the ER or early regions of the Golgi complex.

We did not test the impact of SARS-CoV-2 infection on NF-κB and MAPK signaling, because activation of these pathways has been previously demonstrated (17–19, 51, 76). We assessed the effects of SARS-CoV-2 infection on IFN receptors. Importantly, the effects of infection paralleled those of Nsp14 alone. Over the course of infection, IFNAR1 and IFNGR1 decreased, whereas IFNAR2 remained stable. Of note, after incubation of the virus with the cells, the levels of IFNGR1 detected by immunoblot were higher than for the uninfected cells. The basis for this increase is not clear, but the decrease in IFNGR1 over the course of infection was evident. In addition, as in the transfection studies, BafA1 but not MG132 partly restored IFNAR1 and IFNGR1 levels. These parallels support the relevance of Nsp14 receptor downregulation for SARS-CoV-2 infection. It remains to be determined how broadly/selectively Nsp14 affects cell surface receptors.

Tollip, which we serendipitously found interacts with Nsp14 in co-IP assays, modulates TLR4 signaling and promotes lysosomal degradation of a variety of proteins. Therefore, it was of interest to determine whether Tollip influences the immune modulatory properties of Nsp14. Tollip over-expression inhibits IL-1R, TLR4, or TLR2-mediated NF-κB activation (60, 64, 65, 77). Described mechanisms of Tollip inhibition include binding to the TIR domains of these receptors and binding to the kinase IRAK1 (64, 65). Studies that sought to define the TLR4-interacting regions of Tollip implicated the C2 domain as well as more C-terminal sequences (65). This roughly mirrors the regions that interact with Nsp14. Our data with Tollip point mutants implicate phosphoinositide/LC3 binding in inhibition of Nsp14-mediated NF-κB activation and in NF-κB inhibition in the absence of Nsp14. Our data with the CUE1 and CUE2 mutants indicate that ubiquitin binding is not needed for Tollip inhibition of Nsp14-mediated or Nsp14-independent NF-κB activation. Prior studies have defined mutations in the C2 domain, including mutation K150E, that disrupt binding to phosphoinositides and LC3 (67, 78). Notably, K150 is adjacent to the LC3 binding mutant WYSL-AAAA that we tested, and K150E exhibits reduced capacity to block LPS signaling (78).

Tollip also regulates trafficking of a variety of membrane-associated receptors, suggesting possible relevance to Nsp14 downregulation of IFNAR1 and IFNGR1. For example, IL-1β triggers the ubiquitination of interleukin-1 receptor type 1 (IL-1R1), and this allows interaction with Tollip via its ubiquitin binding CUE domain, leading to the trafficking of IL-1R1 to late endosomes for degradation (60). Tollip also promotes the degradation of TGF-β type I receptor (TβRI) (59) and directs select aberrant membrane proteins in the ER for degradation via the lysosome (61). Suggesting that Tollip is relevant to the Nsp14-mediated downregulation of IFNAR1 and IFNGR1, siRNA knockdown of Tollip in the context of SARS-CoV-2 infection reduced the loss of IFNAR1 and IFNGR1. Rescue of IFNGR1 levels was more apparent, but for IFNAR1, Tollip knockdown had a clear effect at 24 hours post-infection, and a more modest effect was evident at 36 hours post-infection. A prior study reported that Tollip promotes ACE2 degradation and impairs SARS-CoV-2 infection (79). Therefore, it was important to assess the effects of Tollip knockdown in our assays. We did not detect enhanced infection upon Tollip knockdown. This may reflect saturating levels of ACE2 in the A549 cells engineered to express ACE2 that we used for these studies. Interestingly, we detected a modest decrease in viral N protein levels that was most evident at 24 hours post-infection. This corresponded to a modest but not statistically significant decrease in viral RNA. While we cannot exclude some impact of this impaired viral gene expression on IFNAR1 and IFNGR1 downregulation, the effects are sufficiently modest that they likely do not explain the effects of Tollip knockdown.

Cumulatively, our data highlight the contrasting effects of Nsp14, which result in pro-inflammatory innate immune pathway activation and simultaneous downregulation of IFNα and IFNγ signaling (Fig. S5). It is striking that the anti-IFN receptor activities of our Nsp14 mutants paralleled the effects on NF-κB and MAPK signaling, such that activators of NF-κB and MAPK are downregulators of IFNAR1 and IFNGR1. Although the underlying mechanism remains unclear, this suggests a common activity of Nsp14 underpinning these diverse effects.

Limitations of this study include the reliance on transfection-based approaches to define the effects on cellular signaling pathways. The relative contribution of Nsp14 to the activation of these pathways in the context of infection needs further clarification. With regard to Tollip, while it can counteract the effects of Nsp14 on NF-κB activation, analysis of Tollip mutants does not clearly distinguish between Tollip-Nsp14 interaction versus more general effects of Tollip on NF-κB signaling as a mechanism of inhibition. In the context of SARS-CoV-2 infection, the presence of Tollip contributes to IFNAR1 and IFNGR1 downregulation, suggesting a role of the Nsp14-Tollip interaction in receptor downregulation. As with NF-κB activation, it remains to be determined whether and how Nsp14-host protein interactions relate to the effects of N7-MTase mutations. Finally, it seems probable that the mechanisms that activate NF-κB and MAPK and promote IFNAR1 and IFNGR1 downregulation should impact other pathways. The full breadth of effects of Nsp14 on cellular signaling pathways warrants further analysis.

## MATERIALS AND METHODS

### Cell lines and viruses

HEK293T (ATCC, CRL-3216), HEK293/hTLR4A-MD2-CD14 (Invivogen, 293-htlr4md2cd14), Huh7 (a generous gift from the Gordan laboratory at the University of California at San Francisco), A549-ACE2 (a generous gift from the García-Sastre laboratory at the Icahn School of Medicine at Mount Sinai), and VeroE6 (ATCC, CCL-81) cells were maintained in Dulbecco’s modified Eagle’s medium (Corning) with 10% fetal bovine serum (Gibco) at 37°C and 5% CO_2_. Cell treatments included bafilomycin A1 (Invivogen tlrl-baf1), MG132 (Sigma-Aldrich M7449), LPS (Invivogen tlrl-pb5lps), human IFN-γ (PeproTech 300-02-100ug), and Universal Type I IFN (Human IFN-Alpha Hybrid Protein) (PBL Assay Science 11200-1). SARS-CoV-2 isolate USA-WA1/2020 was kindly provided by the García-Sastre laboratory at Icahn School of Medicine at Mount Sinai. Virus stocks were prepared on Vero E6 cells.

### Plasmids

Cloned SARS-CoV-2 Nsp14 (a kind gift from the Krogan laboratory at the University of California at San Francisco) was used to generate wild-type and mutant Nsp14 with an N-terminal HA tag in the mammalian expression plasmid pCAGGS. Human Tollip and IFNGR1 were obtained from GenScript (catalog number OHU02397D) and Sino Biological (catalog number HG10338-CF), respectively. These were cloned with an N-terminal Flag tag in pCAGGS. Primers used for cloning are provided in Table S1. Reporter plasmids used include an NF-κB-responsive firefly luciferase reporter plasmid (pGL4.32 [luc2P/NF-κB RE/Hygro]) (Promega), an IFN-stimulated response element firefly luciferase reporter plasmid (pGL4.45 [luc2P/ISRE/Hygro]) (Promega), a gamma-activated sequence firefly luciferase reporter plasmid (pGL4 [luc2P/GAS RE Hygro]) (Promega), and a constitutively expressing *Renilla* luciferase reporter plasmid (pRL-TK) (Promega).

### DNA transfection and dual luciferase assays

DNA transfection was performed in 96-well plates using Lipofectamine 2000 (Invitrogen catalog number 11668500) according to the manufacturer’s instructions. For the reporter gene assays, at 24 hours post-transfection, cells were stimulated with 100 ng LPS for the NF-κB luciferase reporter, 1,000 IU/mL of universal IFNα for the ISRE luciferase reporter, 500 IU/mL of human interferon-gamma (IFN-γ) for the gamma activated sequence reporter for 18 hours, and lysates were prepared using Passive lysis buffer (Promega). Firefly and *Renilla* luciferase activities were determined with the Dual luciferase reporter assay system (Promega E1960) and an Agilent BioTek Cytation C10 Confocal Imaging Reader. Each transfection was performed in triplicate, and each experiment was repeated at least twice, yielding similar results.

### Co‐immunoprecipitation assays

HEK293T cells were transfected in 6-well plates by using Lipofectamine 2000. After 48 hours incubation, the cells were washed once with phosphate-buffered saline (PBS), lysed in NP40 lysis buffer (50 mM Tris-HCl [pH 8], 280 mM NaCl, 0.5% NP-40, 0.2 mM EDTA, 2 mM EGTA, glycerol 10%, protease inhibitors [Sigma-Aldrich catalog number 11836170001) and centrifuged at 21,130 rcf in an Eppendorf 5424 R microcentrifuge for 10 minutes at 4°C. The resulting supernatants were considered whole-cell extracts. Whole cell extracts were incubated with anti-HA magnetic beads (Pierce catalog number 88836) for 45 minutes at 4°C. A portion of the supernatant was saved as input material. The beads were washed five times with NP40 lysis buffer.

### Immunoblotting

Used were Invitrogen Bolt 4% to 12%, Bis-Tris gels (catalog number NW04120BOX), PVDF membrane (Sigma-Aldrich catalog number 03010040001), 5% blocking buffer (Bio-Rad catalog number 1706404), and Western Chemiluminescent HRP substrate (Perkin Elmer catalog number NEL104001EA). Imaging was performed with a ChemiDoc MP Imaging System (Bio-Rad). Antibody information is provided in Table S2.

### Deglycosidase treatments

Cell lysates were treated with Endo H (New England Biolabs catalog number P0702L) or PNGase F (New England Biolabs catalog number P0704S).

### RNA isolation and quantitative reverse transcription PCR

RNA was isolated using the DirectZol RNA kit (Zymo Research R2052) and reverse transcribed with the SuperScript IV VILO Master Mix with ezDNase Enzyme (catalog number 11766050). Real-time qPCR was conducted using PerfeCTa SYBR Green FastMix (catalog number 101414-272). Quantitative PCR reactions were performed with a Bio-Rad CFX Opus 96 qPCR machine. Relative expression of target mRNAs was normalized to β-actin mRNA, and the fold differences were calculated using the threshold cycle (ΔΔCT) method. Primers used for qPCR are listed in Table S3.

### Immunofluorescence assay

Huh7 cells grown on coverslips were transfected with plasmid DNA for 24 hours. Cells were fixed with 4% paraformaldehyde in PBS with 1 mM CaCl_2_ and 1 mM MgCl_2_ (PBS-CM) for 15–30 minutes at room temperature (RT). Fixed cells were washed twice with PBS-CM and permeabilized for 10 minutes with 0.1% Triton X-100 in PBS-CM at RT. After blocking with 4% goat serum (MP Biomedicals, 0219135680) in PBS supplemented with 0.5% BSA and 0.15% glycine (PBG) for 1 hour at RT, cells were incubated with the primary antibodies in blocking buffer for 1 hour at RT, followed by three washes with PBS for 5 minutes each and incubation with the secondary antibodies for 1 hour at RT. The cells were washed three times with PBS, and the coverslips were mounted with ProLong Glass Antifade Mountant with NucBlue Stain (Thermo Fisher Scientific P36981). Stained cells were examined under a Zeiss LSM980 confocal laser scanning microscope. Images were analyzed with Imaris software (imaris.oxinst.com/), counting at least 50 cells per sample.

### siRNA gene silencing

A549-ACE2 cells were seeded in 6-well plates at a density of 100,000 cells/well. The next day, cells were transfected with 50 nM of Tollip SMARTpool (Dharmacon catalog number L-016930-00-0005) or NON-TARGETplus Non-targeting Control siRNA (Dharmacon, catalog number D-001810-01-05) using Lipofectamine RNAiMAX (Invitrogen, 13778-150). Twenty-four hours post-siRNA transfection, cells were infected with SARS-CoV-2.

## Data Availability

The data described in this study will be made available without restriction upon request.

## SUPPLEMENTAL MATERIAL

Supplemental material mbio.01071-25-s0001.pdf Fig. S1-S5; Tables S1-S3.

ASM does not own the copyrights to Supplemental Material that may be linked to, or accessed through, an article. The authors have granted ASM a non-exclusive, world-wide license to publish the Supplemental Material files. Please contact the corresponding author directly for reuse.
