# Supplementary material for: SARS-CoV-2 Nsp14 binds Tollip and activates pro-inflammatory pathways while downregulating interferon-α and interferon-γ receptors
Source: mBio. 2025 Jun 25;16(8):e01071-25. doi: 10.1128/mbio.01071-25 (PMC12345183; doi:10.1128/mbio.01071-25)
Supplement: Supplemental material — Fig. S1-S5; Tables S1-S3. [file mbio.01071-25-s0001.pdf]

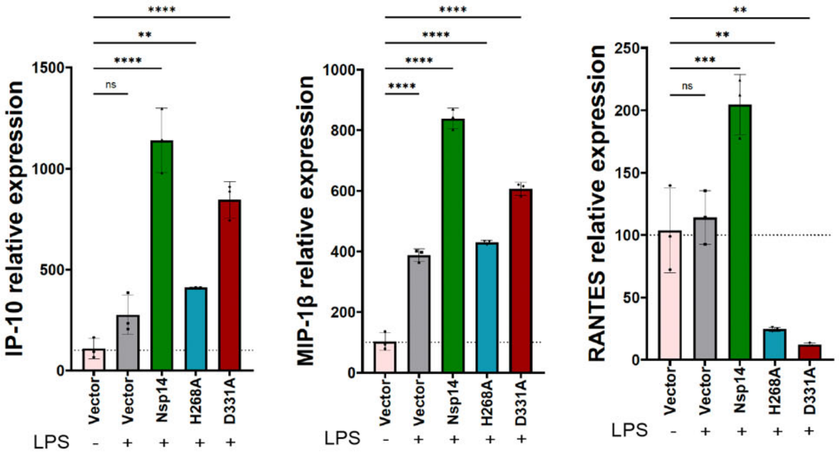

Fig. S1. Expression levels of IP-10, MIP-1β and RANTES as determined by RT-qPCR in cells transfected with empty vector (Vector), Nsp14 and the indicated Nsp14 mutants. Cells were mock-treated or treated with LPS, as indicated. Error bars represent mean ± SD (n = 3). One way ANOVA was used to determine statistical significance ($P < 0.01$ = ∗∗, $P < 0.0001$ = ∗∗∗∗, not significant = ns).

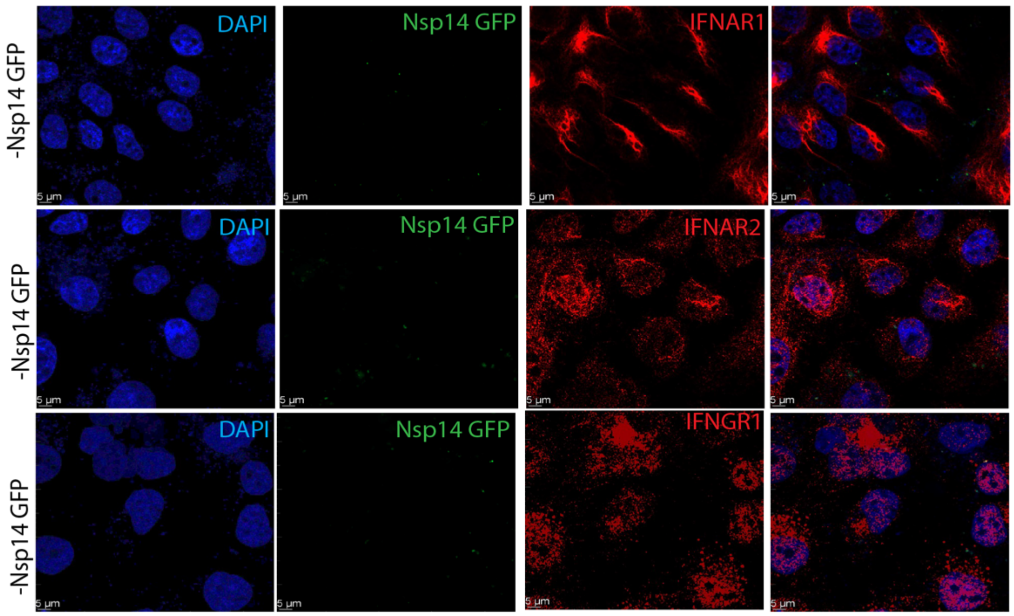

Fig. S2. Empty vector control transfections to assess impact on IFNAR1 and IFNGR1 levels.
Confocal laser scanning microscopy image of indicated interferon receptor expression level in the presence of empty vector transfected Huh7 cells. Blue, DAPI (nuclei); Green, absence of signal due to absence of Nsp14-GFP; Red, IFNAR1, IFNAR2 or IFNGR1.

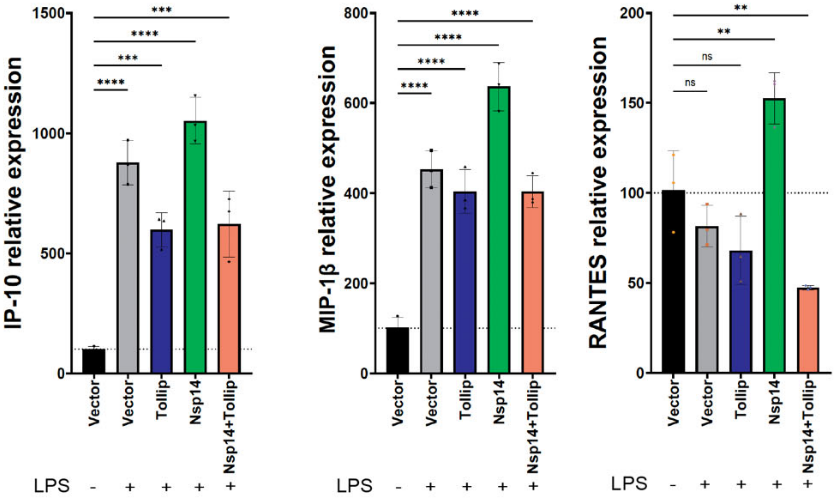

Fig. S3. **Effect of Tollip on chemokine expression in the presence of Nsp14.**
IP-10, MIP-1β and RANTES mRNA levels in in HEK293 cells that express TLR4, MD2 and CD14 transfected with empty vector (Vector), Tollip and/or Nsp14 plasmids, in the absence or presence of LPS, as indicated. Error bars represent mean ± SD (n = 3). One way ANOVA was used to determine statistical significance (P ≤ 0.01 = ∗∗, P < 0.001 = ∗∗∗, P < 0.0001 = ∗∗∗∗, not significant = ns).

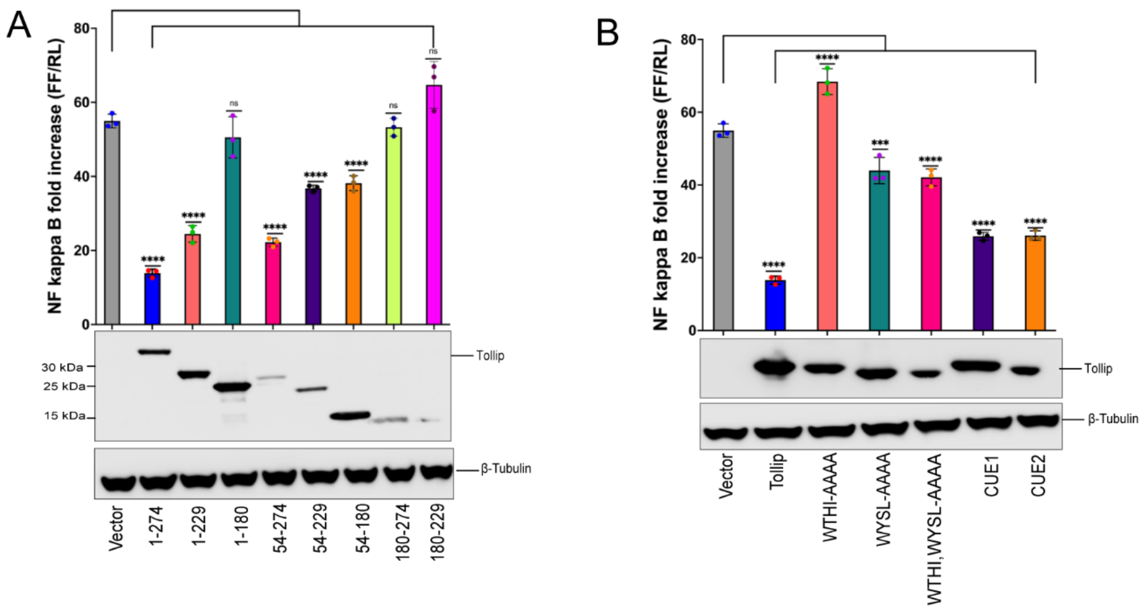

Fig. S4. **Tollip counteracts LPS-mediated NF-κB activation in the absence of Nsp14.**
A. Domain mapping studies. HEK293-hTLR4-MD2-CD14 cells were transfected with an NF-κB-firefly luciferase reporter, a constitutively expressing *Renilla* luciferase reporter and the indicated expression plasmids. At 24 h post-transfection, cells were treated with LPS for 18 h and used for dual luciferase reporter assay. Firefly luciferase activity was normalized to *Renilla* luciferase activity. Data are reported as fold increase relative to a mock-treated, empty vector control. Error bars represent mean ± SD (n = 3). One way ANOVA was used to determine statistical significance ($P < 0.0001$ = ∗∗∗∗, not significant = ns). Cell lysates were analyzed by western blot. β-tubulin served as a loading control.
B. Tollip point mutant studies. An NF-κB-firefly luciferase reporter assay was performed as in A but with the indicated expression plasmids. ($P < 0.001$ = ∗∗∗, $P < 0.0001$ = ∗∗∗∗). Cell lysates were analyzed by immunoblot. β-tubulin served as a loading control.

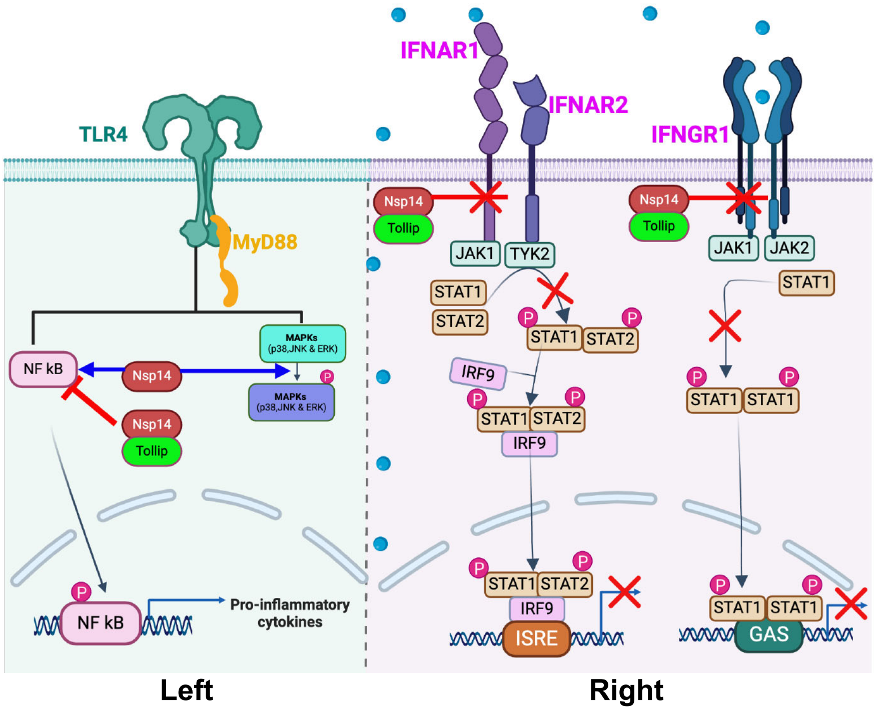

Fig. S5. **Nsp14 effects on innate immune signaling.**

Left. Toll-like receptor 4 (TLR4) is depicted associated with signaling molecule MyD88. As indicated, signaling through TLR4, as occurs when it is activated by LPS, leads to NF-κB and MAP kinase (MAPK) signaling. Nsp14 expression stimulates both NF-κB, leading to its nuclear accumulation and function as a transcription factor, and MAPK signaling through TLR4, as indicated by the blue arrows. This activation requires Nsp14 N7-MTase activity. Tollip interacts with Nsp14 and impairs activation of NF-κB. Whether interaction between Nsp14 and Tollip is required for the inhibition by Tollip remains to be determined.

Right. The heterodimeric IFN alpha receptor consisting of IFNAR1and IFNAR2 and the heterodimeric IFN gamma receptor consisting of IFNGR1and IFNGR2 (not labeled because it was not measured in this study) are depicted at the plasma membrane. Key steps in the signaling pathways are also illustrated. IFNα/β or IFNγ are depicted as blue circles associating with the IFN receptors. This activates IFNAR associated Jak1 and Tyk2 tyrosine kinases, which tyrosine phosphorylate STAT1 and STAT2. Phosphorylated, dimerized STAT1 and STAT2 associate with IRF9 and translocate to the nucleus to activate promoters with IFN stimulated response elements (ISRE). Similarly, IFNGR signaling activates Jak1 and Jak2, leading to tyrosine phosphorylated STAT1 homodimers that translocate to the nucleus to activate promoters with IFN gamma activated sequence (GAS) elements. Nsp14 downregulates IFNAR1 and IFNGR1 leading to blocks in the signaling pathways. This downregulation requires Nsp14 N7-MTase activity. Signaling steps demonstrated to be inhibited by this study are indicated by red Xs. Nsp14 is shown interacting with Tollip. Whether interaction between Nsp14 and Tollip is required for the downregulation of IFNAR1 and IFNGR1 by Tollip remains to be determined, but the presence of Tollip appears to facilitate IFNAR1 and IFNGR1 downregulation in SARS-CoV-2 infected cells. Ps in the red circles represent phosphorylation. Curved light blue dashes indicate the nuclear membrane.

Table S1| Sequences of primers used for cloning in this study

Gene	Primers
Nsp14	Fwd (5′ to 3′): GATTATGCCGCGGCCGCTGCTGAAAATGTAACGGGCTTG
	Rev (5′ to 3′): CCGATGTCCTGCTTAGCTAGCTCACTACTGCAATCGGG
Nsp14 $_{D90A/E92A}$	Fwd (5′ to 3′): CCTGGATAGGCTTCGCAGTTGCAGGTTGCCATGCAACCC
	Rev (5′ to 3′): CGGGTTGCATGGCAACCTGCAACTGCGAAGCCTATCC
Nsp14 $_{E191A}$	Fwd (5′ to 3′): TCTGTGGGCACATGGCTTCGCACTTACTTCTATGAAATAC
	Rev (5′ to 3′): AAGTATTTCATAGAAGTAAGTGCGAAGCCATGTGCCCACAG
Nsp14 $_{H268A}$	Fwd (5′ to 3′): CTTTACTGCCAAGTTGCCGGGAACGCACACGTGGCCTC
	Rev (5′ to 3′): GCCACGTGTGCGTTCCCGGCAACTTGGCAGTAAAGATCG
Nsp14 $_{D273A}$	Fwd (5′ to 3′): TGGCCTCCTGCGCAGCGATAATGACGAGGTGCTTGG
	Rev (5′ to 3′): GCCAAGCACCTCGTCATTATCGCTGCGCAGGAGGC
Nsp14 $_{N306A}$	Fwd (5′ to 3′): CCCATAATCGGTGACGAACTTAAAATTGCCGCTGCAT
	Rev (5′ to 3′): GCATGCAGCGGCAATTTTAAGTTCGTCACCGATTATGGG
Nsp14 $_{D331A}$	Fwd (5′ to 3′): GGATAAGTTTCCTGTGCTCCACGCCATCGGCAACCCTAAG
	Rev (5′ to 3′): TTAGGGTTGCCGATGGCGTGGAGCACAGGAAACTTATCCG
Nsp14 $_{\Delta Nsp10bs}$	Fwd (5′ to 3′): GATTATGCCGCGGCCGCTTACCAAGTTAATGGCTAC
	Rev (5′ to 3′): CCGATGTCCTGCTTAGCTAGCTCACTACTGCAATCGGG
Nsp14 $_{ExoN}$	Fwd (5′ to 3′): GATTATGCCGCGGCCGCTGCTGAAAATGTAACGGGCTTG
	Rev (5′ to 3′): CCGATGTCCTGCTTAGCTAGCTCAAAAGCACTCGTGCACGG
Nsp14 $_{N7\text{-}MTase.}$	Fwd (5′ to 3′): GATTATGCCGCGGCCGCTGTTAAGCGGGTCGATTGGACTA
	Rev (5′ to 3′): CCGATGTCCTGCTTAGCTAGCTCACTACTGCAATCGGG
Nsp14-GFP	Fwd (5′ to 3′): GATTATGCCGCGGCCGCTGCTGAAAATGTAACGGGCTTG
Nsp14-GFPOV	Fwd(5′to3′): GATTGCAGCAATTGTCTGGAGGAAGCGGAGGCTCCGGCA
Nsp14-GFPOV	Rev (5′ to 3′): CTTGCTCACCATGCCGGAGCCTCCGCTTCCTCCAGACAAT
Nsp14-GFP R	Rev (5′ to 3′): GCTAGCGGATCCTTACTTGTACAGCTCGTCCA
Tollip	Fwd(5′to3′):GATTATGCCGCGGCCGCT ATGGCGACCACCGTCAGCACTCA
	Rev (5′ to 3′): CCGATGT CCTGCTTAGCTAGCTCAGCCTATGGCTCCTCC
Tollip $_{1\text{-}229}$	Fwd (5′ to 3′): AAAAAAGCGGCCGCA ATGGCGACCACCGTCAGCA
	Rev (5′ to 3′): AAAAAAGCTAGCTCAACAGCGGGGCTGGGCGT

Table S1| Sequences of primers used for cloning in this study

Gene	Primers
Tollip $_{1-180}$	Fwd (5′ to 3′): AAAAAAGCGGCCGCAATGGCGACCACCGTCAGCA
	Rev (5′ to 3′): AAAAAA GCTAGCTCACACCATGGCAGCTGGAAGC
Tollip $_{54-274}$	Fwd (5′ to 3′): AAAAAAGCGGCCGCACGACTGAACATCACGGTGGTA
	Rev (5′ to 3′): AAAAAAGCTAGCCTATGGCTCCTCCCCCAT
Tollip $_{54-229}$	Fwd (5′ to 3′): AAAAAAGCGGCCGCACGACTGAACATCACGGTGGTA
	Rev (5′ to 3′): AAAAAA GCTAGCTCAACAGCGGGGCTGGGCG
Tollip $_{54-180}$	Fwd (5′ to 3′): AAAAAAGCGGCCGCACGACTGAACATCACGGTGGTA
	Rev (5′ to 3′): AAAAAA GCTAGCTCACACCATGGCAGCTGGAAGC
Tollip $_{180-274}$	Fwd (5′ to 3′): AAAAAAGCGGCCGCAGTGATGCCACCCCAGC
	Rev (5′ to 3′): AAAAAAGCTAGCCTATGGCTCCTCCCCCAT
Tollip $_{180-229}$	Fwd (5′ to 3′): AAAAAAGCGGCCGCAGTGATGCCACCCCAGC
	Rev (5′ to 3′): AAAAAAGCTAGCTCAACAGCGGGGCTGGGCGT
Tollip$_{WTHI/AAAA}$	Fwd (5′ to 3′): GGACGACCGCATTGCAGCGGCAGCAGCAACCATCCCGGA
	Rev (5′ to 3′): CTCCGGGATGGTTGCTGCTGCCGCTGCAATGCGGTCGTC
Tollip $_{WYSL/AAAA}$	Fwd (5′ to 3′): GGTGGAGGACAAGGCGGCGGCGGCGAGCGGGAGGCAG
	Rev (5′ to 3′): CTGCCTCCCGCTCGCCGCCGCCGCCTTGTCCTCCACC
Tollip $_{M240A/F241A}$	Fwd (5′ to 3′): AGCCATCCAGGACGCTGCCCCCAACATGGACCAGGAG
	Rev (5′ to 3′): TCCTGGTCCATGTTGGGGGCAGCGTCCTGGATGGCTTTC
Tollip$_{L267A/L268A}$	Fwd (5′ to 3′): GATTATGCCGCGGCCGCT ATGGCGACCACCGTCAGCACTC
	Rev (5′ to 3′): GCTCCTCCCCCATCTGAGCGGCGGAGTTGATGGC
IFNGR1	Fwd (5′ to 3′): GATTATGCCGCGGCCGCTATGGCTCTCCTCTTTCTCCTAC
	Rev (5′ to 3′): CCGATGTCCTGCTTAGCTAGCTCATGAAAATTCTTTGGAAT

Table S2 | Antibodies used in this study

Protein	Antibodies
Tollip	Abcam, (ab187198), anti Tollip Rabbit polyclonal
p38	Cell Signaling Technology (9212S), anti p38 Rabbit monoclonal
Phospho-p38	Cell Signaling Technology (4511S) anti Phospho-p38 Rabbit monoclonal
ERK	Cell Signaling Technology (9102S), anti ERK Rabbit polyclonal
Phospho-ERK	Cell Signaling Technology (9101S), anti Phospho-ERK Rabbit polyclonal
JNK	Cell Signaling Technology (9252S), Anti JNK Rabbit polyclonal
Phospho-JNK	Cell Signaling Technology (9151S), anti Phospho-JNK Rabbit polyclonal
IFNAR1	Abcam (ab124764), anti IFNAR1 Rabbit monoclonal (Western blot)
IFNAR1	Abclonal, (A18594), anti IFNAR1 Rabbit polyclonal (IFA)
IFNAR2	Abcam (ab56070), anti IFNAR2 Rabbit polyclonal
IFNGR1	R & D Systems (MAB6731) anti IFNGR1 Mouse monoclonal
STAT1	BD Biosciences (610186), anti STAT1 Mouse monoclonal
P-STAT1	Cell Signaling Technology (9167S), anti Phospho-STAT1 Rabbit monoclonal
GAPDH	Thermo Fisher Scientific (MA5-15738), anti GAPDH Mouse monoclonal
β Tubulin	Sigma (T8328), anti β Tubulin Mouse monoclonal
Flag-tag	Sigma-Aldrich (F3165), anti-Flag Mouse monoclonal
HA-tag	Sigma-Aldrich (H3663) anti HA Mouse monoclonal
Flag-tag	Sigma-Aldrich(F7425), anti Flag Rabbit polyclonal
HA-tag	Invitrogen (71-5500), anti HA Rabbit polyclonal
Anti-mouse IgG	Cell Signaling Technology (7076), Anti-mouse IgG, HRP-linked Antibody
Anti-rabbit IgG	Cell Signaling Technology (7074), Anti-rabbit IgG, HRP-linked Antibody
Alexa Fluor™ 647	Invitrogen (A-21236), Goat anti-Mouse, Secondary Antibody
Alexa Fluor™ 594	Invitrogen (A-32740), Goat anti-Rabbit Secondary Antibody

Table S3 | Sequences of primers for qPCR analysis of this study

Gene	Primers
IL 1 β	Fwd (5′ to 3′): CTCGCCAGTGAAATGATGGCT
	Rev (5′ to 3′): GTCGGAGATTCGTAGCTGGAT
IL6	Fwd (5′ to 3′): CCTGAACCTTCCAAAGATGGC
	Rev (5′ to 3′): TTCACCAGGCAAGTCTCCTCA
IL8	Fwd (5′ to 3′): GGCCAAGAGAATATCCGAAC
	Rev (5′ to 3′): AGGCACAGTGGAACAAGGAC
IL 10	Fwd (5′ to 3′): CAAATGAAGGATCAGCTGGACAA
	Rev (5′ to 3′): GCATCACCTCCTCCAGGTAAAAC
IP10	Fwd (5′ to 3′): TCCCATCACTTCCCTACATG
	Rev (5′ to 3′): TGAAGCAGGGTCAGAACATC
MIP 1 β	Fwd (5′ to 3′): CGTGTATGACCTGGAACTGAACTG
	Rev (5′ to 3′): TCCCTGAAGACTTCCTGTCTCTGA
RANTES	Fwd (5′ to 3′): TTGCCAGGGCTCTGTGACCA
	Rev (5′ to 3′): AAGCTCCTGTGAGGGGTTGA
β -Actin	Fwd (5′ to 3′): ACTGGAACGGTGAAGGTGAC
	Rev (5′ to 3′): GTGGACTTGGGAGAGGACTG
SARS CoV-2 N	Fwd (5′ to 3′): GGGGAACTTCTCCTGCTAGAAT
	Rev (5′ to 3′): CAGACATTTTGCTCTCAAGCTG
